# A latent class analysis of patterns of tobacco and cannabis use in Australia and their health‐related correlates

**DOI:** 10.1111/dar.13614

**Published:** 2023-02-13

**Authors:** Carmen C. W. Lim, Janni K. Y. Leung, Shannon Gravely, Coral Gartner, Tianze Sun, Vivian Chiu, Jack Y. C. Chung, Daniel Stjepanović, Jason Connor, Roman W. Scheurer, Wayne Hall, Gary C. K. Chan

**Affiliations:** ^1^ National Centre for Youth Substance Use Research, Faculty of Health and Behavioural Sciences The University of Queensland Brisbane Australia; ^2^ School of Psychology The University of Queensland Brisbane Australia; ^3^ NHMRC Centre of Research Excellence on Achieving the Tobacco Endgame, School of Public Health, Faculty of Medicine The University of Queensland Brisbane Australia; ^4^ Department of Psychology University of Waterloo Waterloo Canada; ^5^ Discipline of Psychiatry, Faculty of Medicine The University of Queensland Brisbane Australia; ^6^ Queensland Centre for Mental Health Research The Park Centre for Mental Health Brisbane Australia; ^7^ Queensland Alliance for Environmental Health Sciences The University of Queensland Brisbane Australia

**Keywords:** cannabis, cigarettes, joints, marijuana, tobacco

## Abstract

**Introduction:**

The shifting landscape in Australia's tobacco and cannabis policies and emerging new products and modes of administration may increase experimentation and the risks of addiction to these drugs.

**Methods:**

We analysed cross‐sectional data from the 2019 National Drug Strategy and Household Survey (*n* = 22,015) of Australians aged 14 and above. Latent class analysis was used to identify distinct groups based on types of tobacco and cannabis products used. The socio‐demographic, health‐rated correlates and past‐year substance use of each latent class was examined.

**Results:**

A four‐class solution was identified: co‐use of tobacco and cannabis (2.4%), cannabis‐only (5.5%), tobacco‐only (8.0%) and non‐user (84.0%). Males (odds ratio [OR] range 1.5–2.9), younger age (OR range 2.4–8.4), moderate to high psychological distress (OR range 1.3–3.0), using illicit substances in the last year (OR range 1.41–22.87) and high risk of alcohol use disorder (OR range 2.0–21.7) were more likely to be in the tobacco/cannabis use classes than non‐users. Within the co‐use class, 78.4% mixed tobacco with cannabis and 89.4% had used alcohol with cannabis at least once.

**Discussion and Conclusions:**

Approximately 16% of respondents used tobacco or cannabis, or both substances, and no major distinct subgroups were identified by the use of different product types. Mental health issues and the poly‐substance use were more common in the class who were co‐users of cannabis and tobacco. Existing policies need to minimise cannabis and tobacco‐related harms to reduce the societal burden associated with both substances.

## 1 INTRODUCTION

Tobacco and cannabis are the most commonly used psychoactive substances after alcohol in Australia [1]. These substances have different health impacts and population‐level disease burdens. In 2018, 8.6% of the total disease burden was attributable to tobacco smoking while cannabis use accounted for 0.3% of the total disease burden in Australia [2]. A key prevention strategy to reduce the disease burden is to understand the key characteristics of the consumers of single or both substances and the factors related to their patterns of consumption.

Co‐use of tobacco and cannabis is prevalent in many countries including Australia [3, 4], with co‐use practices differing by country and region [5]. Co‐use of tobacco and cannabis can involve sequential use, where tobacco and cannabis are used separately within the same use episode, or simultaneous use when both substances are mixed or used separately during the same occasion [6]. The health effects of co‐using cannabis and tobacco may be more harmful than the use of either substance alone [7, 8] from the additive risks of toxicant exposure [9, 10].

In Australia, the regulatory environment for tobacco and cannabis is rapidly evolving. To reduce tobacco smoking, Australia has implemented plain packaging, health warnings on tobacco products, tobacco advertising bans [11] and annual increments in tobacco excise [12]. Many individuals made smoking‐related behaviour changes in response to increasing cigarette prices by quitting smoking, reducing consumption, changing to lower‐priced brands, using loose tobacco or using e‐cigarettes [13]. As for cannabis, non‐therapeutic cannabis is regulated as an illicit substance federally but each state sets penalties for its use, possession and cultivation [14]. In 2016, the Australian federal government approved the prescription, dispensing, cultivation and manufacture of medicinal cannabis products [15]. The Australia Capital Territory implemented new rules in 2020 to legalise the possession of small amounts of cannabis for personal use [16]. These rules along with emerging cannabis products and modes of administration [17] may increase experimentation and the risk of addiction.

The study aims to use latent class analysis (LCA) to identify distinct groups within the Australian population based on 11 tobacco and cannabis products used. In addition, the socio‐demographic, general health, psychological distress and substance use correlate for each latent class is identified. The main benefit of the LCA approach is that it can group individuals according to the use of a wide range of tobacco and cannabis products, hence, allowing a more nuanced characterisation of how different products might be used in combination [18]. Since an individual's pattern of tobacco and cannabis product use can range from not using to using more than one tobacco and/or cannabis product (e.g., using cigarettes only or using joints, edibles and cigarettes, etc.), the LCA approach is preferred over the dichotomisation of (11! = 39,916,800) variables.

Studies examining the patterns of poly‐substance use using LCA have found distinct subpopulations of tobacco and cannabis users that used different products in combination [19, 20, 21]. For example, Evans‐Polce and colleagues identified five unique classes of substance use behaviour including a class on ‘hookah and marijuana’ among college students [19]. Based on these studies, it is expected that there will be at least one group who co‐used both cannabis and tobacco products.

## 2 METHODS

### 2.1 Data

The 2019 National Drug Strategy Household Survey (NDSHS) is a multi‐stage, stratified survey that is conducted triennially to assess the attitudes and behaviours in relation to drug use in Australia. The sampling frame includes respondents aged 14 and above in residential households across Australia. A total of 22,015 people (mean age 45.3 years, 42% male) were surveyed in 2019, with a response rate of 49%. Full details of the survey are described elsewhere [1].

### 2.2 Measures

### 2.3 Cannabis products used

The four cannabis product variables analysed were: (i) joint; (ii) bong; (iii) edibles; and (iv) vapes. Respondents were asked if they had used cannabis in the last 12 months. If so, they were asked *‘how have you used Marijuana/Cannabis?’*. Options to this question were: ‘*Smoked as Joints*’, ‘*Smoked from a bong/pipe*’, *‘Inhaled through a vaporising device’* and ‘*By eating it*’. Respondents could report more than one form of use. Four dichotomous variables were created for each method of use (yes vs. no for each variable).

### 2.4 Tobacco products used

The seven tobacco product variables analysed were: (i) manufactured cigarettes; (ii) roll‐your‐own cigarettes; (iii) e‐cigarettes; (iv) cigarillos; (v) cigars; (vi) water pipe tobacco; and (vii) other tobacco products. Respondents were asked *‘how often, if at all, do you now smoke manufactured cigarettes, roll‐your‐own cigarettes, cigarillos, cigars, water pipe tobacco, or pipe tobacco?’*. Response options to this question were ‘*Daily*’, ‘*At least weekly* (*but not daily*)’, ‘*Less often than weekly*’ or ‘*Not at all*’. Six dichotomous variables were created for each tobacco product with any endorsement ‘*daily or at least weekly*’ or ‘*less often than weekly*’ to the frequency question coded as ‘*yes*’ to the specific tobacco product. The NDSHS also asked ‘*Which, if any, of the following products* [*chewing tobacco, snuff, snus, bidis*] *have you ever used, and which have you used in the last 12 months?’*. Four dichotomous variables were created with endorsement to ‘used in last 12 months’ coded as ‘*yes*’. Finally, the e‐cigarette question was based on ‘*How often, do you currently use electronic cigarette?*’. Response options to this question were ‘*Daily*’, ‘*At least weekly* (*but not daily*)’, ‘*At least monthly* (*but not weekly*)*’*, *‘Less than monthly’*, *‘I used to use them but no longer use’*, *‘I only tried them once or twice’* or ‘*Not at all*’. Endorsement to the first three responses was coded ‘yes’ to e‐cigarette use. Low prevalence (<0.5%) tobacco products (chewing tobacco, snuff, snus, bidis and pipe tobacco) were grouped as ‘*other tobacco product*’.

### 2.5 Socio‐demographic correlates

The following variables were used in the analysis: sex (*‘male’*, *‘female’*), marital status (*‘never married’*, *‘divorced*, *separated or widowed’*, *‘currently married including de‐facto’*), highest education attainment (*‘high school or less’*, *‘certificate or diploma’*, *‘bachelor degree or higher’*), remoteness of the area of dwelling (*‘major cities’*, *‘inner regional’*, *‘outer regional*, *remote or very remote area’*), country of birth (*‘Australia’*, *‘not Australia’*) and current employment status (*‘not in labour force’*, *‘unemployed’*, *‘currently employed’*). Household annual income were coded by dividing the income distribution into quartiles (*‘low income’*, *‘low‐average’*, *‘high‐average’*, *‘high income’*).

### 2.6 Self‐reported general health and psychological distress

Self‐rated general health was measured using the question ‘*In general*, *would you say your health is ‘excellent’*, *‘very good’*, *‘good’*, *‘fair’*, *‘poor’*?’. The Kessler psychological distress scale (K10), a 10‐item questionnaire based on respondents' anxiety and depressive symptoms in the past month, was used to assess the levels of psychological distress—(*‘low’*, *‘moderate’*, *‘high or very high level’*) [22].

### 2.7 Substance use in the last 12 months

Alcohol use status was assessed using the Alcohol Use Disorders Identification Test Consumption short‐form (AUDIT‐C) scale [23]. The AUDIT‐C is an alcohol screen that identifies hazardous drinking patterns and a potential alcohol use disorder (AUD). There was a slight difference in how the questions and responses in NDSHS were worded compared to AUDIT‐C. Based on an approximation used by prior studies [24, 25], a similar scoring system was derived and NDSHS respondents were classified as ‘no or low risk’ for AUD (total score <4 for male and <3 for female), ‘high risk’ for AUD (total score > = 4 for male and > =3 for female). A comparison between AUDIT‐C and NDSHS 2019 questions are provided in Table S1, Supporting Information. Respondents were also asked if they had used any of the following illicit drugs for recreational purposes in the last 12 months: ecstasy, meth/amphetamine, cocaine, hallucinogens, inhalants and opioids. Low prevalence (<2%) drugs were excluded: heroin, gamma‐hydroxybutyrate, synthetic cannabis, novel psychoactive substances, ketamine, tranquilisers, non‐medical steroid use and other opioids (methadone).

### 2.8 Analysis

Descriptive statistics were computed to estimate the weighted prevalence of tobacco and cannabis products. LCA is a statistical method for identifying unobservable or latent groups of respondents that are similar based on a set of observed categorical variables [26]. LCA was used to identify patterns of tobacco and cannabis use by the 11 product type variables (joints, bong, edibles, vapes, manufactured cigarettes, roll‐your‐own cigarettes, e‐cigarettes, cigarillos, cigars, water pipe tobacco and other tobacco products). LCA was conducted using MPLUS version 8.6 [27]. Survey weights were applied when estimating the prevalence of each latent class. Missing values in any of the tobacco or cannabis product variables were handled using full information maximum likelihood estimation in MPlus.

We fitted a series of LCA models (2–5 classes). The optimal number of classes was determined using a range of information criteria (Akaike Information Criteria [AIC], Bayesian Information Criteria [BIC], Sample size adjusted BIC) and likelihood ratio test (Vuong‐Lo–Mendell–Rubin adjusted likelihood ratio test) and classification diagnostics (entropy and average posterior probabilities). Lower values in information criterion were preferred as it indicates a better balance between model fit and model parsimony. The Vuong‐Lo–Mendell–Rubin adjusted likelihood ratio test is also used to compare models with *k* classes versus *k*‐1 classes. A significant *p*‐value suggests that the model with *k* classes fit the data better than models with *k*‐1 classes [28]. Entropy values approaching 1 indicate a clear delineation of classes [29], although it was argued that entropy is a poor diagnostic to select the optimal number of class [30]. Average posterior probability was estimated for membership in each class, given class assignment. Values that are close to 1 suggest that there is a high degree of certainty about the true class membership for each individual. The unconditional probabilities and BCH weights for all 22,015 respondents were generated in MPlus, and this dataset was merged with the original NDSHS dataset which contains socio‐demographic, health‐rated and substance‐use correlates.

The correlates, patterns of substance use and health factors of the class membership were examined using multivariate multinomial logistic regressions in STATA17.0 [31]. All class membership comparisons were weighted by BCH weights. The missing values for the covariates ranged from 0.4% for marital status to 31.0% for household income (see Table S5). Prior to multinomial logistic regression, multiple imputations by chained equations (20 imputations) was performed to impute the missing values by assuming data are missing at random. A complete case analysis was conducted to evaluate the sensitivity of the multiple imputations.

All analyses were weighted to adjust for differential probabilities of selection within households and to adjust for non‐response rates and to match the samples to the population sociodemographic distribution. Significance level, *α*, was set at 0.0029 (*α* = 0.05/17) to adjust for multiple comparisons. The codes for the analysis can be found at https://github.com/clim072/patterns-of-tobacco-and-cannabis-use.

## 3 RESULTS

### 3.1 Prevalence of cannabis and tobacco use

Table 1 shows the prevalence of cannabis and tobacco use by product type. In the total sample, joints (9.4%) and manufactured cigarettes (11.2%) were the most common cannabis and tobacco products, respectively. Among current cannabis users, the most prevalent cannabis products were joints (82.3%) while the most prevalent tobacco products were manufactured cigarettes (32.6%). The most prevalent cannabis and tobacco products respectively among current tobacco users were joints (28.1%) and manufactured cigarettes (78.0%).

**TABLE 1:** Prevalence of cannabis and tobacco product use.

Products	Total sample (*n* = 22,015)				Among current cannabis users^b^ (*n* = 2273)				Among current tobacco users^c^ (*n* = 3221)			
	*n*	%^a^	95% CI		*n*	%^a^	95% CI		*n*	%^a^	95% CI	
I. Cannabis	2273	11.5	11.0	12.1	‐	‐	‐		‐	‐	‐	
Joint	1825	9.4	8.9	9.9	1825	82.3	80.3	84.3	809	28.1	26.1	30.2
Bong	1623	8.2	7.7	8.7	1623	71.8	69.4	74.1	780	26.9	24.9	28.9
Edible	1105	5.6	5.2	6.0	1105	48.8	46.2	51.4	480	16.3	14.7	18.0
Vape	140	0.8	0.6	1.0	140	7.1	5.7	8.5	58	2.2	1.5	2.8
II. Tobacco/nicotine	3221	14.6	14.0	15.2	‐	‐	‐		‐	‐	‐	
Manufactured cigarettes	2420	11.2	10.7	11.7	717	32.6	30.1	35.0	2420	78.0	76.2	79.8
Roll‐your‐own cigarettes	1250	6.0	5.6	6.4	547	25.6	23.3	28.0	1250	41.7	39.5	43.9
E‐cigarettes	393	2.0	1.8	2.3	146	7.8	6.3	9.3	233	7.9	6.7	9.1
Cigarillos	328	1.5	1.3	1.7	101	4.6	3.4	5.7	328	10.2	8.9	11.5
Cigars	155	0.8	0.6	0.9	56	2.5	1.7	3.2	155	5.3	4.2	6.3
Water pipe tobacco	116	0.6	0.4	0.7	79	3.5	2.7	4.4	116	4.1	3.3	5.0
Other tobacco products^d^	210	1.1	0.9	1.2	87	3.9	2.9	4.9	210	4.6	3.7	5.5
Chewing tobacco	107	0.5	0.4	0.7	34	1.6	1.0	2.2	59	2.1	1.5	2.7
Pipe tobacco	68	0.3	0.2	0.4	36	1.3	0.8	1.9	68	2.0	1.4	2.6
Snuff	56	0.3	0.2	0.4	23	1.4	0.8	2.1	31	1.3	0.8	1.8
Snus	54	0.3	0.2	0.4	24	1.2	0.7	1.7	29	1.1	0.6	1.5
Bidis	48	0.3	0.2	0.4	14	0.8	0.35	1.3	29	1.2	0.7	1.7

Abbreviation: CI, confidence interval.

^a^ Weighted prevalence.

^b^ Assessed among those with current cannabis use (use in the last year).

^c^ Assessed among current tobacco smokers (excluding e‐cigarette use) ‐ comprised of daily and non‐daily smokers.

^d^ Chewing tobacco, pipe tobacco, snuff, snus, bidis.

### 3.2 Latent class analysis

Table S2 shows the model fit statistics for a 2–5 class solution based on the latent class model on patterns of cannabis and tobacco use. The AIC, BIC, Sample Size Adjusted BIC statistics showed the lowest value for the 5‐class solution but the difference between the 4‐ and 5‐class solution is very small, suggesting both solutions fit the data well. The LRM‐LRT indicated that the 5‐class solution did not fit the data better than the 4‐class solution. Entropy and average posterior probabilities from the 4‐class solution were close to 1, indicating clear class separation. Given that the classes from the 4‐class solution were clearly interpretable and meaningful, the 4‐class solution was chosen as the most optimal.

**FIGURE 1:**
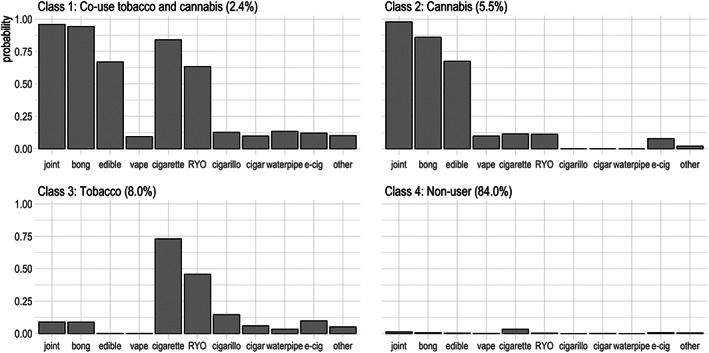
Item response probabilities for each class. RYO, roll‐your‐own.

Figure 1 shows the item‐response probabilities for each tobacco and cannabis item. Item response probabilities greater than or equal to 0.5 are considered to be high probabilities [26]. Class 1 was the co‐use tobacco and cannabis group (2.4%). This group was characterised by high probabilities of using joints (probability, *p* = 0.96), bongs (*p* = 0.94), edibles (*p* = 0.67), manufactured cigarettes (*p* = 0.84) and roll‐your‐own cigarettes (*p* = 0.63). Class 2 was the cannabis‐only group (5.5%). Respondents in this group had a high probability of using joints (*p* = 0.98), bongs (*p* = 0.86) and edibles (*p* = 0.67), however, very few vaped cannabis. Class 3 consists of respondents who predominantly used tobacco (8.0%). Respondents in this class had a high probability of using manufactured cigarettes (*p* = 0.73) and a moderate probability of using roll‐your‐own cigarettes (*p* = 0.46). Respondents in Class 4 had near zero probabilities of using tobacco and cannabis (84.0%).

### 3.3 Associations between latent class membership and socio‐demographics, health‐related correlates and past‐year substance use using non‐user class as the reference group

Table 2 shows the multinomial logistic regression model using class membership as the outcome and socio‐demographic correlates, general health, psychological distress, past‐year substance use and risk of AUD as the predictors. Non‐user was used as the reference for the outcome while females, persons aged 60+, currently married, high household income, major cities, bachelor or higher education, living outside of Australia, currently employed, excellent general health, low psychological distress, did not endorse past‐year substance use, and no or low risk for AUD were specified as the reference groups for the predictors.

**TABLE 2:** Associations between latent class memberships and socio‐demographics, health‐rated correlates and history of past‐year substance use with non‐use as reference group (Class 4).

	Co‐use tobacco and cannabis (Class 1)				Cannabis (Class 2)				Tobacco (Class 3)			
	OR	99.7% CI		*F*‐statistic, *p*‐value	OR	99.7% CI		*F*‐statistic, *p*‐value	OR	99.7% CI		*F*‐statistic, *p*‐value
Sex (ref: female)												
Male	2.9*	1.8	4.8	*F* = 43.7*, *p* < 0.001	1.5*	1.2	1.9	*F* = 30.4*, *p* < 0.001	1.7*	1.4	2.1	*F* = 61.9* *p* < 0.001
Age group, years (ref: 60+)												
14–29	8.4*	2.4	29.6	*F* = 9.2*, *p* < 0.001	4.9*	2.8	8.6	*F* = 27.2*, *p* < 0.001	2.4*	1.5	3.9	*F* = 55.2*, *p* < 0.001
30–39	7.8*	2.3	26.9		4.9*	2.8	8.4		4.1*	2.8	6.2	
40–59	7.0*	2.2	22.3		4.0*	2.4	6.7		4.0*	2.8	5.6	
Marital status (ref: currently married)												
Never married	1.9	1.0	3.5	*F* = 5.2, *p* = 0.006	1.9*	1.4	2.7	*F* = 23.0*, *p* < 0.001	1.6*	1.2	2.2	*F* = 24.7*, *p* < 0.001
Divorced/separated/widowed	1.8	0.9	3.9		1.7*	1.2	2.5		1.8*	1.4	2.4	
Household income (ref: 1st quartile: high)												
2nd quartile (high‐average)	2.1	1.0	4.5	*F* = 4.6*, *p* = 0.003	1.2	0.9	1.8	*F* = 2.0, *p* = 0.110	1.7*	1.2	2.5	*F* = 12.1*, *p* < 0.001
3rd quartile (low average)	2.1	0.9	4.5		1.3	0.9	1.9		1.6*	1.2	2.3	
4th quartile (low)	2.6*	1.1	6.3		1.3	0.8	2.1		2.1*	1.4	3.1	
Remoteness (ref: major cities)												
Inner regional	1.1	0.6	2.0	*F* = 2.7, *p* = 0.071	1.2	0.9	1.6	*F* = 3.6, *p* = 0.027	1.4*	1.1	1.8	*F* = 16.3*, *p* < 0.001
Outer regional, remote or very remote	1.6	0.9	3.0		1.3	0.9	1.8		1.6*	1.2	2.1	
Highest education attainment (ref: bachelor or higher)												
High school or less	1.6	0.8	3.2	*F* = 5.1, *p* = 0.006	1.0	0.7	1.4	*F* = 2.3, *p* = 0.098	2.3*	1.7	3.3	*F* = 30.3*, *p* < 0.001
Certificate or diploma	2.1*	1.0	4.1		1.2	0.9	1.6		2.2*	1.6	3.0	
Country of birth (ref: outside of Australia)												
Australia	1.4	0.7	2.9	*F* = 1.7, *p* = 0.188	1.1	0.8	1.5	*F* = 1.6, *p* = 0.202	0.9	0.7	1.1	*F* = 2.1, *p* = 0.144
Employment (ref: currently employed)												
Not in labour force	0.9	0.4	2.0	*F* = 3.4, *p* = 0.034	0.7	0.5	1.1	*F* = 2.2, *p* = 0.106	0.8	0.6	1.2	*F* = 6.4*, *p* = 0.002
Unemployed	1.7	0.9	3.3		0.9	0.6	1.5		1.4	1.0	1.9	
General health (ref: excellent)												
Very good	1.1	0.5	2.4	*F* = 7.6*, *p* < 0.001	1.2	0.9	1.7	*F* = 2.3, *p* = 0.059	2.1*	1.3	3.3	*F* = 36.7*, *p* < 0.001
Good	2.2	1.0	4.8		1.3	0.9	1.8		3.7*	2.3	5.8	
Fair	3.4*	1.4	8.5		1.6	1.0	2.7		5.2*	3.1	8.7	
Poor	3.3	0.7	15.9		1.6	0.6	4.1		6.7*	3.5	12.9	
Psychological distress (ref: K10 low)												
K10—moderate	1.6	0.9	2.8	*F* = 15.8*, *p* < 0.001	1.3*	1.0	1.8	*F* = 11.6*, *p* < 0.001	1.0	0.8	1.3	*F* = 7.6*, *p* = 0.001
K10—high to very high	3.0*	1.7	5.3		1.6*	1.2	2.3		1.4*	1.1	1.9	
Past‐year substance use (ref: no)												
Ecstasy	3.5*	1.4	8.5	*F* = 17.1*, *p* < 0.001	3.2*	1.6	6.4	*F* = 26.6*, *p* < 0.001	1.6	0.7	3.9	*F* = 2.9, *p* = 0.089
Meth/amphetamine	21.7*	5.3	88.6	*F* = 42.3*, *p* < 0.001	9.5*	2.5	36.3	*F* = 24.9*, *p* < 0.001	11.0*	3.3	36.8	*F* = 35.0*, *p* < 0.001
Cocaine	8.9*	4.4	18.0	*F* = 86.2*, *p* < 0.001	4.9*	3.0	8.0	*F* = 92.9*, *p* < 0.001	3.2*	1.8	5.6	*F* = 37.3*, *p* < 0.001
Hallucinogens	10.2*	3.1	33.7	*F* = 33.4*, *p* < 0.001	8.8*	3.2	24.0	*F* = 41.1*, *p* < 0.001	2.2	0.6	8.0	*F* = 3.0, *p* = 0.084
Inhalants	1.8	0.5	5.6	*F* = 2.0, *p* = 0.152	1.5	0.6	3.7	*F* = 2.1, *p* = 0.150	1.2	0.5	3.2	*F* = 0.4, *p* = 0.491
Opioids	4.2*	2.0	8.8	*F* = 32.7*, *p* < 0.001	1.6	0.8	3.0	*F* = 4.7, *p* = 0.030	2.0*	1.2	3.3	*F* = 17.7*, *p* < 0.001
AUDIT‐C (Risk of alcohol use disorder) (ref: no or low risk)												
High risk	5.3*	2.9	9.5	*F* = 71.5*, *p* < 0.001	3.8*	2.9	4.9	*F* = 229.3*, *p* < 0.001	2.2*	1.8	2.7	*F* = 117.6*, *p* < 0.001

Abbreviations: AUDIT‐C, Alcohol Use Disorders Identification Test Consumption short‐form; CI, confidence interval; OR, odds ratio.

* Significant at the 0.0029 level, two sided test.

#### 3.3.1 Socio‐demographic correlates

Males, respondents aged less than 60, and those with low household income were more likely to be classified in the co‐use class (Class 1) (*p* < 0.05). Meanwhile, males, respondents aged less than 60, and never having married or divorced were likely to be assigned to the cannabis‐only class (Class 2) (*p* < 0.001). Males, respondents aged less than 60, those who were never married or divorced, not having a high household income, not living in major cities and obtained less than a bachelor degree were more likely to be in the tobacco‐only class (Class 3) (*p* < 0.05).

#### 3.3.2 General health and psychological distress

Respondents reporting fair level of general health were more likely to be in the co‐use class (Class 1) (*p* < 0.001). Respondents reporting all but excellent level of general health were more likely to be in the tobacco‐only class (Class 3) (*p* < 0.001). Respondents who reported high to very high levels of psychological distress were likely to be in co‐use and tobacco‐only class (*p* < 0.001 to *p* = 0.001) while respondents reported moderate to very high levels of psychological distress were likely to be in cannabis‐only class (*p* < 0.001).

#### 3.3.3 Past‐year substance use and risk of AUD

Respondents that reported past‐year use of ecstasy, methamphetamine, cocaine, hallucinogens or opioids were likely to be in the co‐use class (Class 1) (*p* < 0.001). Meanwhile, respondents reporting past‐year ecstasy, methamphetamine, cocaine or hallucinogen use were more likely to be assigned to the cannabis‐only class (Class 2) (*p* < 0.001). Individuals reporting past‐year methamphetamine, cocaine or opioid use were more likely to be in the tobacco‐only class (Class 3) (*p* < 0.001). Finally, respondents with high risk of AUD were likely to be assigned to all three substance classes (*p* < 0.001). The prevalence of correlates and other factors for each latent class can be found in Table S3.

Sensitivity analyses were performed by running the multinomial logistic regression on complete cases (listwise deletion of cases) (see Table S4). While the results remained largely similar between complete case analysis and multiple imputation analysis, the results from the complete case analysis had larger standard errors and wider confidence intervals, which may result in less statistical power than multiple imputation results.

### 3.4 Associations between latent class membership and socio‐demographics, health‐rated correlates, and past‐year substance use using cannabis‐only or tobacco‐only class as reference the group

Table 3 shows the multinomial logistic regression model using class membership as the outcome. The first column of the table used cannabis class as the reference for the outcome while the second column used tobacco‐only class as the reference for the outcome. The reference groups for the predictors remained the same.

**TABLE 3:** Associations between latent class memberships and correlates, health outcomes and history of past‐year substance use with tobacco and cannabis class as reference group.

	Cannabis (Class 2) as reference group				Tobacco (Class 3) as reference group			
	OR	99.7% CI		*F*‐statistic, *p*‐value	OR	99.7% CI		*F*‐statistic, *p*‐value
Sex (ref: female)								
Male	1.9*	1.2	3.2	*F* = 15.3*, *p* < 0.001	1.7*	1.0	2.8	*F* = 9.8*, *p* = 0.002
Age group, years (ref: 60+)								
14–29	1.7	0.4	6.6	*F* = 0.6, *p* = 0.601	3.5	0.9	12.8	*F* = 3.9, *p* = 0.009
30–39	1.6	0.4	6.0		1.9	0.5	6.7	
40–59	1.7	0.5	6.0		1.8	0.5	5.7	
Marital status (ref: currently married)								
Never married	1.0	0.5	1.8	*F* = 0.1, *p* = 0.947	1.1	0.6	2.2	*F* = 0.2, *p* = 0.791
Divorced/separated/widowed	1.1	0.5	2.4		1.0	0.5	2.2	
Household income (ref: 1st quartile: high)								
2nd quartile (high‐average)	1.7	0.8	3.6	*F* = 2.2, *p* = 0.083	1.2	0.6	2.7	*F* = 0.3, *p* = 0.819
3rd quartile (low average)	1.6	0.7	3.5		1.3	0.5	2.9	
4th quartile (low)	2.0	0.9	4.8		1.2	0.5	3.1	
Remoteness (ref: major cities)								
Inner regional	0.9	0.5	1.7	*F* = 0.9, *p* = 0.391	0.8	0.4	1.5	*F* = 0.6, *p* = 0.539
Outer regional, remote or very remote	1.3	0.7	2.5		1.0	0.5	1.9	
Highest education attainment (ref: bachelor or higher)								
High school or less	1.6	0.8	3.2	*F* = 2.8, *p* = 0.063	0.7	0.3	1.5	*F* = 2.0, *p* = 0.136
Certificate or diploma	1.7	0.9	3.4		1.0	0.5	2.0	
Country of birth (ref: outside of Australia)								
Australia	1.2	0.6	2.6	*F* = 0.6, *p* = 0.430	1.6	0.7	3.4	*F* = 3.2, *p* = 0.073
Employment (ref: currently employed)								
Not in labour force	1.2	0.4	3.0	*F* = 2.6, *p* = 0.076	1.0	0.4	2.5	*F* = 0.4, *p* = 0.662
Unemployed	1.8	0.8	3.8		1.2	0.6	2.5	
General health (ref: excellent)								
Very good	0.9	0.4	2.0	*F* = 4.2, *p* = 0.020	0.5	0.2	1.3	*F* = 1.2, *p* = 0.291
Good	1.7	0.8	3.8		0.6	0.2	1.4	
Fair	2.1	0.8	5.5		0.6	0.2	1.8	
Poor	2.1	0.4	11.6		0.5	0.1	2.5	
Psychological distress (ref: K10—low)								
K10—moderate	1.2	0.7	2.2	*F* = 4.4, *p* = 0.013	1.6	0.9	2.9	*F* = 6.9*, *p* = 0.001
K10—high to very high	1.8	1.0	3.3		2.1*	1.2	3.9	
Past‐year substance use (ref: no)								
Ecstasy	1.1	0.5	2.3	*F* = 0.1, *p* = 0.783	2.1	0.8	5.4	*F* = 5.7, *p* = 0.017
Meth/amphetamine	2.3*	1.0	5.0	*F* = 9.9*, *p* = 0.002	2.0	0.8	4.7	*F* = 5.4, *p* = 0.020
Cocaine	1.8	0.9	3.6	*F* = 7.2, *p* = 0.007	2.8*	1.3	5.9	*F* = 17.4*, *p* < 0.001
Hallucinogens	1.2	0.5	2.6	*F* = 0.3, *p* = 0.568	4.7*	1.3	17.7	*F* = 12.3*, *p* < 0.001
Inhalants	1.1	0.4	3.1	*F* = 0.1, *p* = 0.696	1.4	0.4	5.3	*F* = 0.6, *p* = 0.444
Opioids	2.6*	1.2	5.5	*F* = 14.4*, *p* < 0.001	2.1	1.0	4.4	*F* = 8.0, *p* = 0.005
AUDIT‐C (Risk of alcohol use disorder)								
High risk	1.4	0.8	2.6	*F* = 2.6, *p* = 0.106	2.4*	1.3	4.4	*F* = 19.0*, *p* < 0.001

Abbreviations: AUDIT‐C, Alcohol Use Disorders Identification Test Consumption short‐form; CI, confidence interval; OR, odds ratio.

* Significant at the 0.0029 level, two sided test.

Males, those reporting methamphetamine and opioid use in the past year were likely to be assigned to the co‐use class compared to the cannabis‐only class (Class 2) (*p* < 0.001 to *p* = 0.002). Those experiencing high to very high psychological distress, reporting cocaine, hallucinogen use or endorse high‐risk AUD were more likely to be in the co‐use class compared to tobacco‐only class (Class 3) (*p* < 0.001 to *p* = 0.002).

### 3.5 Simultaneous use of other substances with cannabis and tobacco on at least one occasion

Table 4 shows among those in Class 1 (co‐use tobacco and cannabis), 78.4% of respondents mixed cannabis and tobacco. They were also likely to use other substances simultaneously in the past year. These included alcohol (89.4%), ecstasy (36.6%), cocaine (30.7%), hallucinogens (25.5%), methamphetamine (22.3%) and opioids (20.6%).

**TABLE 4:** Simultaneous use of other substances among co‐use tobacco and cannabis class in the past year.

	Co‐use tobacco and cannabis (Class 1)		
	%	95% CI	
I. Marijuana/cannabis and tobacco mixed	78.4	75.8	81.1
II. Simultaneous use of other substances			
Alcohol	89.4	87.5	91.3
Ecstasy	36.6	33.5	39.8
Cocaine, crack	30.7	27.6	33.7
Hallucinogens, LSD, magic mushrooms	25.5	22.5	28.5
Methamphetamine^a^	22.3	19.6	24.9
Pain killers, pain relievers and opioids^a^	20.6	18.1	23.2
Ketamine	13.2	10.9	15.5
Tranquillisers, sleeping pills^a^	8.9	7.1	10.6
Heroin	3.2	2.1	4.3
GHB	2.1	1.2	3.0
Sniffing petrol, glue, aerosols, solvents	2.0	1.1	2.8
Methadone, buprenorphine^a^	1.8	1.1	2.5
Kava	1.6	1.0	2.3
Steroids^a^	1.4	0.6	2.2

Abbreviation: CI, confidence interval.

^a^ For non‐medical use.

## 4 DISCUSSION

This study of patterns of tobacco and cannabis use in Australia identified four distinct groups of individuals: co‐use of tobacco and cannabis class (2.4% from the sample), cannabis‐only class (5.5%), tobacco‐only class (8.0%) and a non‐user class (84.0%). We used an LCA approach to explore potential subgroups of individuals who may differ according to the tobacco and cannabis product types used, however, these major distinct subgroups were not identified. Our analysis revealed predominate product types used within each of the latent classes. Our co‐use class was characterised by high probabilities of using joints, bongs, edibles, manufactured cigarettes and roll‐your‐own cigarettes. The cannabis‐only class was characterised by high probabilities of joints, bongs and edible use while the tobacco‐only class was characterised by high probabilities of using manufactured cigarettes and moderate probability of using roll your own. Combustible forms of tobacco and cannabis were common but other routes of administration via inhalation (e.g., vaping) were less common in our study, potentially reflecting the strict regulations imposed on the sales of nicotine and vaping devices [32]. The shifting landscape of tobacco and cannabis warrants ongoing surveillance through national surveys, sales data and other forms of surveillance such as online cryptomarkets.

The identified classes in this study are consistent with prior literature in showing an overlap between tobacco and cannabis use [7, 33, 34]. Although shared genetic liabilities are a possible explanation for the four distinct classes of tobacco and cannabis, currently there is lack of replication studies to confirm the genes and chromosomal regions related to cannabis [35] and tobacco use [36]. These genome‐wide association studies also utilised crude measurements on cannabis such as lifetime cannabis use [37, 38]. The exact mechanism remains unclear as there are other explanations such as shared risk factors through peer influences, availability of products and regulatory environment.

In terms of socio‐demographics correlates, our study found males, respondents aged less than 60, and having high to very high psychological distress were associated with all tobacco and cannabis classes. The proportion of respondents in the co‐use class was small (2.4%); however, young people (aged 14–29) had an 8‐fold increased odds of being assigned to this class compared to non‐users. The odds of past year use of methamphetamine, opioids, cocaine and hallucinogens were also higher in the co‐use class than tobacco or cannabis‐only classes. Young people are susceptible to risk taking [39] and their substance patterns could be influenced by peer influence, stressful events and employment [7]. This suggests the need to enhance mental health support among these groups through community campaigns, peer‐support or workplace interventions.

In addition, the present study also showed high rates of combining alcohol (89.4%) with cannabis use among those co‐using both tobacco and cannabis. The high rates of simultaneous use of other substances among co‐users may compromise the success of quit attempts [7]. The effects of both drugs may be intensified when combined, and hence increase the chance of negative outcomes, such as drunk driving [40]. Physicians should also assess and address alcohol use while treating tobacco and cannabis addictions especially in young people since alcohol use in this age group is extremely common [1].

Our study found that 78% of the respondents in the co‐use class had mixed cannabis with tobacco. The 2018 International Tobacco Control Policy Evaluation Study also showed that co‐users in Australia were more likely to mix tobacco with cannabis [4]. Co‐use may also complicate cessation treatment when patients are dependent on both nicotine and cannabis use [4, 41]. McClure and colleagues found cannabis use increased by 50% during tobacco cessation quit attempts; similarly, tobacco use increased by 62% during cannabis cessation [42]. Evidence on dual interventions (e.g., providing nicotine replacement therapy or medication along with behavioural treatment) to reduce co‐use have been shown to reduce cannabis use but there is no clear effect on tobacco cessation [43]. Guidance counsellors, general practitioners, and psychologists could also help the affected individual to understand their substance use behaviour and to introduce coping strategies.

The strengths of the current study were its large sample from a population‐based survey that comprehensively measured a range of substances and relevant socio‐demographic variables and the use of LCA to simultaneously consider how tobacco and cannabis products were used in combination. There were several limitations inherent in the present work. Although the estimates can be broadly generalised to the Australian population, the NDSHS has limitations pertaining to cross‐sectional surveys. The NDSHS do not sample persons in institutional settings such as hospitals, nursing homes, and those lacking permanent addresses. Interviews were not conducted in languages other than English. The surveys also relied on the retrospective recall of respondents' experience with cannabis and tobacco. Our findings are unlikely to be generalisable to high‐risk or specific sub‐populations. The current analysis has only examined the latest NDSHS dataset, future research could consider examining changes in the demographics associated with class membership over time. Although multiple imputation were used, there are limitations because some variables have up to 31% of missing values, and there could be other variables that were not measured by the survey and so we were not able to include a comprehensive list of all possible variables that may help predict the missing values. Nevertheless, multiple imputations can reduce biases from missing data and previous comparative research has shown that it can be robust even in the presence of large percentages of missing values [44].

## 5 CONCLUSIONS

Approximately 16% of the Australian population used either tobacco, cannabis or both substances in 2019. A small proportion co‐used both substances and these were young people with higher levels of psychological distress who are more likely to simultaneously use other illicit substances. Existing policies need to minimise cannabis and tobacco related harms to reduce societal burden associated with both substances.

## AUTHOR CONTRIBUTIONS

Carmen C. W. Lim: Conceptualisation, methodology, formal analysis, writing—original draft preparation. Gary C. K. Chan: Conceptualisation, methodology, supervision, writing—review and editing. Janni K. Y. Leung: Conceptualisation, methodology, supervision, writing—review and editing. Shannon Gravely, Coral Gartner, Daniel Stjepanović, Wayne Hall, Jack Y. C. Chung: Supervision, writing—review and editing. Tianze Sun, Vivian Chiu, Jack Y. C. Chung, Roman W. Scheurer: Writing—review and editing. Each author certifies that their contribution to this work meets the standards of the International Committee of Medical Journal Editors.

## FUNDING INFORMATIONS

Carmen C. W. Lim is supported by a National Health Medical Research Council (NHMRC) of Australia Postgraduate Scholarship (APP2005317), The University of Queensland Living Stipend and Tuition Scholarship and a National Centre for Youth Substance Use Research top‐up scholarship. Janni K. Y. Leung is supported by an NHMRC Investigator Fellowship (APP2010008) and The University of Queensland development fellowship. Gary C. K. Chan is supported by an NHMRC Investigator Fellowship (APP1176137). The National Centre for Youth Substance Use Research is supported by Commonwealth funding from the Australian Government provided under the Drug and Alcohol Program. The funding bodies had no role in the study design, collection, analysis or interpretation of the data, writing the manuscript, or the decision to submit the paper for publication. The authors would like to acknowledge the Australian Institute of Health and Welfare for providing access to the 2019 National Drug Strategy Household Survey through the Australian Data Archive (ADA Dataverse) and the NDSHS respondents.

## CONFLICT OF INTEREST

The authors declare no known competing financial interest or personal relationships that could influence the work reported in this paper.

## ETHICS STATEMENT

This project has been reviewed by the University of Queensland Human Research Ethics Committee and deemed to be exempt from ethics review (ref: 2021/HE001006).

## Supporting information


**Table S1.** A comparison between AUDIT‐C and 2019 NDSHS questions, and the scoring systems used to derive the risk levels of alcohol use status.
**Table S2.** Fit statistics from latent class models on cannabis and tobacco use in the total sample (*N* = 22,015).
**Table S3.** Sample characteristics by latent class membership.
**Table S4.** Associations between latent class memberships and socio‐demographics, health rated correlates and history of past‐year substance use with non‐user as reference group (Class 4).
**Table S5.** Number and proportion of missing values.
