# Supplementary material for: A latent class analysis of patterns of tobacco and cannabis use in Australia and their health‐related correlates
Source: Drug Alcohol Rev. 2023 Feb 13;42(4):815–26. doi: 10.1111/dar.13614 (PMC10947067; doi:10.1111/dar.13614)
Supplement: Supplementary file 1 — Table S1. A comparison between AUDIT‐C and 2019 NDSHS questions, and the scoring systems used to derive the risk levels of alcohol use status. Table S2. Fit statistics from latent class models on cannabis and tobacco use in the total sample (N = 22,015). Table S3. Sample characteristics by latent class membership. Table S4. Associations between latent class memberships and socio‐demographics, health rated correlates and history of past‐year substance use with non‐user as reference group (Class 4). Table S5. Number and proportion of missing values. [file DAR-42-815-s001.docx]

**Table S1: A comparison between AUDIT-C and 2019 NDSHS questions, and the scoring systems used to derive the risk levels of alcohol use status.**

| **AUDIT-C questions** | **Questions in 2019 NDSHS questionnaire** |
| --- | --- |
| How often do you have a drink containing alcohol?   \| Responses \| Scores \| \| --- \| --- \| \| Never \| 0 \| \| Monthly or less \| 1 \| \| 2-4 times / month \| 2 \| \| 2-3 times / week \| 3 \| \| 4+ times / week \| 4 \| | E7. In the last 12 months, how often did you have an alcoholic drink of any kind?   \| Responses \| Scores \| \| --- \| --- \| \| Every day \| 4 \| \| 5 to 6 days a week \| 4 \| \| 3 to 4 days a week \| 3.5 \| \| 1 to 2 days a week \| 2.5 \| \| 2 to 3 days a month \| 2 \| \| About 1 day a month \| 1 \| \| Less often \| 1 \| \| No longer drink \| 0 \| |
| How many standard drinks containing alcohol do you have on a typical day?   \| Responses \| Scores \| \| --- \| --- \| \| 1-2 \| 0 \| \| 3-4 \| 1 \| \| 5-6 \| 2 \| \| 7-9 \| 3 \| \| 10+ \| 4 \| | E14. On a day that you have an alcoholic drink, how many standard drinks do you usually have?   \| Responses \| Scores \| \| --- \| --- \| \| 20 or more standard drinks \| 4 \| \| 16 – 19 standard drinks \| 4 \| \| 13 – 15 standard drinks \| 4 \| \| 11 – 12 standard drinks \| 4 \| \| 9 – 10 standard drinks \| 3.5 \| \| 7 – 8 standard drinks \| 3 \| \| 5 – 6 standard drinks \| 2 \| \| 3 – 4 standard drinks \| 1 \| \| 2 standard drinks \| 0 \| \| 1 standard drink \| 0 \| \| Half a standard drink \| 0 \| |
| How often do you have six or more drinks on one occasion?   \| Responses \| Scores \| \| --- \| --- \| \| Never \| 0 \| \| Less than monthly \| 1 \| \| Monthly \| 2 \| \| Weekly \| 3 \| \| Daily or almost daily \| 4 \| | E16. Please record how often in the last 12 months you have had each of the following number of standard drinks in a day?   \| Responses/ Scores \| Everyday \| 5-6 days /week \| 3-4 days /week \| 1-2 days /week \| 2-3 days /month \| About 1 day /month \| Less often \| Never \| \| --- \| --- \| --- \| --- \| --- \| --- \| --- \| --- \| --- \| \| 20 or more standard drinks/day \| 4 \| 4 \| 3 \| 3 \| 2 \| 2 \| 1 \| 1 \| \| 11 – 19 standard drinks/day \| 4 \| 4 \| 3 \| 3 \| 2 \| 2 \| 1 \| 1 \| \| 7 – 10 standard drinks/day \| 4 \| 4 \| 3 \| 3 \| 2 \| 2 \| 1 \| 1 \| \| 5 – 6 standard drinks/day \| 4 \| 4 \| 3 \| 3 \| 2 \| 2 \| 1 \| 1 \| \| 3 – 4 standard drinks/day \| N/A \| N/A \| N/A \| N/A \| N/A \| N/A \| N/A \| N/A \| \| 1 – 2 standard drinks/day \| N/A \| N/A \| N/A \| N/A \| N/A \| N/A \| N/A \| N/A \| \| Less than 1 standard drink/day \| N/A \| N/A \| N/A \| N/A \| N/A \| N/A \| N/A \| N/A \| \| None \| N/A \| N/A \| N/A \| N/A \| N/A \| N/A \| N/A \| N/A \| |

AUDIT-C, Alcohol Use Disorders Identification Test Consumption short-form, N/A, not applicable; NDSHS, National Drug Strategy Household Survey.

**Table S2. Fit statistics from latent class models on cannabis and tobacco use in the total sample (N= 22,015)**

| **Number of class** | **AIC** | **BIC** | **SSABIC** | **LRM-LRT, p-value** | **Entropy** | **log likelihood** | **Average posterior probability** |
| --- | --- | --- | --- | --- | --- | --- | --- |
| 2 | 60105.9 | 60289.8 | 60216.8 | 16753.4, p<0.001 | 0.955 | -30029.93 | Class 1: 0.973 |
|  |  |  |  |  |  |  | Class 2: 0.991 |
| 3 | 56642.2 | 56922.2 | 56811.0 | 3458.8, p<0.001 | 0.917 |  | Class 1: 0.889 |
|  |  |  |  |  |  | -28286.11 | Class 2: 0.981 |
|  |  |  |  |  |  |  | Class 3: 0.972 |
| 4 | 56120.3 | 56496.2 | 56346.9 | 541.4, p <0.001 | 0.915 |  | Class 1: 0.899 |
|  |  |  |  |  |  | -28013.13 | Class 2: 0.883 |
|  |  |  |  |  |  |  | Class 3: 0.916 |
|  |  |  |  |  |  |  | Class 4: 0.966 |
| 5 | 56021.1 | 56493.0 | 56305.5 | 122.2, p = 0.660 | 0.917 |  | Class 1: 0.836 |
|  |  |  |  |  |  |  | Class 2: 0.844 |
|  |  |  |  |  |  | -27951.54 | Class 3: 0.675 |
|  |  |  |  |  |  |  | Class 4: 0.878 |
|  |  |  |  |  |  |  | Class 5: 0.972 |

AIC, Akaike Information Criteria; BIC, Bayesian Information Criteria, LMR-LRT, Lo-Mendell-Rubin adjusted Likelihood Ratio Test; SSABIC, Sample Size Adjusted Bayesian Information Criteria.

**Table S3. Sample characteristics by latent class membership**

|  | **Total sample** | | | | **Co-use tobacco and cannabis**  **(class 1 - 2.4%)** | | | | **Cannabis-only**  **(class 2 - 5.5%)** | | | | **Tobacco-only**  **(class 3 - 8.0%)** | | | | **Non-users**  **(class 4 - 84.0%)** | | | |
| --- | --- | --- | --- | --- | --- | --- | --- | --- | --- | --- | --- | --- | --- | --- | --- | --- | --- | --- | --- | --- |
|  | % | | 95% CI | | % | | 95% CI | | % | | 95% CI | | % | | 95% CI | | % | | 95% CI | |
| **Sex** |  |  | |  |  |  | |  |  |  | |  |  |  | |  |  |  | |  |
| Males | **49.2** | 48.8 | | 49.6 | **68.5** | 65.6 | | 71.4 | **61.9** | 60.2 | | 63.6 | **58.9** | 57.2 | | 60.6 | **47.3** | 46.9 | | 47.8 |
| Females | **50.8** | 50.4 | | 51.2 | **31.5** | 28.6 | | 34.4 | **38.1** | 36.4 | | 39.8 | **41.1** | 39.4 | | 42.8 | **52.7** | 52.2 | | 53.1 |
| **Age group, years** |  |  | |  |  |  | |  |  |  | |  |  |  | |  |  |  | |  |
| 14-29 | **26.0** | 25.6 | | 26.4 | **52.0** | 48.8 | | 55.2 | **47.7** | 45.9 | | 49.5 | **23.3** | 21.6 | | 25.0 | **24.2** | 23.7 | | 24.6 |
| 30-39 | **17.6** | 17.2 | | 17.9 | **18.9** | 16.6 | | 21.2 | **22.3** | 20.8 | | 23.7 | **20.2** | 18.8 | | 21.5 | **17.0** | 16.7 | | 17.4 |
| 40-59 | **30.3** | 30.0 | | 30.7 | **26.2** | 23.6 | | 28.9 | **24.5** | 23.1 | | 25.9 | **43.0** | 41.2 | | 44.7 | **30.0** | 29.6 | | 30.4 |
| 60+ | **26.1** | 25.8 | | 26.4 | **2.8** | 2.1 | | 3.6 | **5.5** | 4.8 | | 6.2 | **13.6** | 12.6 | | 14.6 | **28.8** | 28.4 | | 29.1 |
| **Marital status** |  |  | |  |  |  | |  |  |  | |  |  |  | |  |  |  | |  |
| Never married | **27.9** | 27.5 | | 28.4 | **58.1** | 55.0 | | 61.3 | **53.5** | 51.7 | | 55.2 | **32.0** | 30.3 | | 33.7 | **25.3** | 24.9 | | 25.8 |
| Divorced/separated/widowed | **12.2** | 12.0 | | 12.5 | **10.7** | 8.8 | | 12.5 | **9.1** | 8.2 | | 10.0 | **16.8** | 15.7 | | 18.0 | **12.2** | 11.9 | | 12.4 |
| Currently married/de facto | **59.8** | 59.4 | | 60.3 | **31.2** | 28.3 | | 34.1 | **37.5** | 35.8 | | 39.1 | **51.2** | 49.4 | | 53.0 | **62.5** | 62.1 | | 63.0 |
| **Household income** |  |  | |  |  |  | |  |  |  | |  |  |  | |  |  |  | |  |
| 1st quartile (high) | **39.4** | 38.8 | | 40.0 | **39.3** | 34.8 | | 43.8 | **48.0** | 45.8 | | 50.3 | **28.6** | 26.4 | | 30.8 | **39.5** | 38.9 | | 40.1 |
| 2nd quartile (high-average) | **15.7** | 15.1 | | 16.3 | **18.8** | 14.8 | | 22.8 | **17.4** | 15.3 | | 19.6 | **19.2** | 17.2 | | 21.2 | **15.3** | 14.6 | | 15.9 |
| 3rd quartile (low-average) | **21.0** | 20.6 | | 21.5 | **20.7** | 15.9 | | 25.5 | **20.6** | 18.3 | | 22.8 | **23.2** | 20.6 | | 25.8 | **20.9** | 20.4 | | 21.5 |
| 4th quartile (low) | **23.9** | 23.3 | | 24.5 | **21.2** | 16.8 | | 25.7 | **14.0** | 11.9 | | 16.0 | **29.0** | 26.4 | | 31.7 | **24.3** | 23.7 | | 24.8 |
| **Remoteness** |  |  | |  |  |  | |  |  |  | |  |  |  | |  |  |  | |  |
| Major cities | **72.5** | 72.2 | | 72.8 | **71.7** | 72.2 | | 75.2 | **73.7** | 72.2 | | 75.2 | **64.4** | 62.7 | | 66.0 | **73.0** | 72.7 | | 73.3 |
| Inner regional | **18.8** | 18.5 | | 19.0 | **18.5** | 17.1 | | 19.9 | **18.5** | 17.1 | | 19.9 | **22.6** | 21.2 | | 24.1 | **18.6** | 18.2 | | 18.9 |
| Outer regional, remote or very remote | **8.7** | 8.5 | | 8.9 | **11.1** | 9.2 | | 13.1 | **7.8** | 6.9 | | 8.6 | **13.0** | 11.9 | | 14.1 | **8.4** | 8.2 | | 8.6 |
| **Highest Education Attainment** |  |  | |  |  |  | |  |  |  | |  |  |  | |  |  |  | |  |
| High school or less | **40.8** | 40.4 | | 41.3 | **39.8** | 36.2 | | 43.4 | **37.0** | 35.1 | | 38.8 | **48.4** | 46.4 | | 50.3 | **40.6** | 40.1 | | 41.1 |
| Certificate or diploma | **29.1** | 28.7 | | 29.5 | **40.3** | 36.9 | | 43.8 | **35.0** | 33.1 | | 36.8 | **36.4** | 34.6 | | 38.2 | **28.0** | 27.5 | | 28.4 |
| Bachelor or higher | **30.1** | 29.6 | | 30.5 | **19.9** | 16.8 | | 23.0 | **28.0** | 26.3 | | 29.8 | **15.2** | 13.5 | | 17.0 | **31.4** | 31.0 | | 31.9 |
| **Country of birth** |  |  | |  |  |  | |  |  |  | |  |  |  | |  |  |  | |  |
| Australia | **72.6** | 72.2 | | 73.1 | **87.7** | 85.1 | | 90.2 | **82.7** | 81.2 | | 84.1 | **76.4** | 74.5 | | 78.3 | **71.4** | 70.9 | | 71.9 |
| Outside of Australia | **27.4** | 26.9 | | 27.8 | **12.3** | 9.8 | | 14.9 | **17.3** | 15.9 | | 18.8 | **23.6** | 21.7 | | 25.5 | **28.6** | 28.1 | | 29.1 |
| **Employment** |  |  | |  |  |  | |  |  |  | |  |  |  | |  |  |  | |  |
| Not in labour force | **28.9** | 28.5 | | 29.3 | **9.9** | 8.0 | | 11.8 | **9.7** | 8.6 | | 10.7 | **23.5** | 21.9 | | 25.1 | **30.9** | 30.5 | | 31.4 |
| Unemployed | **8.8** | 8.5 | | 9.1 | **17.5** | 15.0 | | 20.1 | **10.6** | 9.4 | | 11.8 | **14.5** | 13.0 | | 16.1 | **8.1** | 7.8 | | 8.4 |
| Currently employed | **62.3** | 61.9 | | 62.8 | **72.6** | 69.6 | | 75.6 | **79.7** | 78.2 | | 81.2 | **62.0** | 60.2 | | 63.8 | **61.0** | 60.5 | | 61.4 |
| **General health** |  |  | |  |  |  | |  |  |  | |  |  |  | |  |  |  | |  |
| Excellent | **17.9** | 17.6 | | 18.3 | **9.4** | 7.6 | | 11.3 | **18.7** | 17.2 | | 20.2 | **7.2** | 6.2 | | 8.2 | **18.8** | 18.4 | | 19.2 |
| Very good | **38.6** | 38.2 | | 39.0 | **29.9** | 26.9 | | 33.0 | **40.4** | 38.6 | | 42.2 | **29.4** | 27.8 | | 31.0 | **39.3** | 28.8 | | 39.7 |
| Good | **31.8** | 31.4 | | 32.2 | **41.4** | 38.2 | | 44.6 | **29.8** | 28.2 | | 31.4 | **41.9** | 40.2 | | 43.7 | **31.0** | 30.6 | | 31.4 |
| Fair | **9.7** | 9.4 | | 9.9 | **16.7** | 14.4 | | 19.1 | **9.7** | 8.6 | | 10.7 | **17.0** | 15.7 | | 18.3 | **9.0** | 8.8 | | 9.3 |
| Poor | **2.0** | 1.9 | | 2.1 | **2.5** | 1.6 | | 3.5 | **1.4** | 1.0 | | 1.8 | **4.5** | 3.8 | | 5.1 | **1.9** | 1.8 | | 2.0 |
| **Psychological distress** |  |  | |  |  |  | |  |  |  | |  |  |  | |  |  |  | |  |
| K10 - low | **63.9** | 63.5 | | 64.4 | **34.3** | 31.1 | | 37.4 | **46.5** | 44.7 | | 48.3 | **53.5** | 51.7 | | 55.2 | **66.5** | 66.0 | | 66.9 |
| K10 - moderate | **21.7** | 21.3 | | 22.0 | **26.5** | 23.6 | | 29.5 | **28.0** | 26.4 | | 29.6 | **22.4** | 21.0 | | 23.9 | **21.1** | 20.7 | | 21.4 |
| K10 - high to very high | **14.4** | 14.1 | | 14.7 | **39.2** | 36.0 | | 42.3 | **25.5** | 23.9 | | 27.1 | **24.1** | 22.6 | | 25.6 | **12.5** | 12.2 | | 12.8 |
| **Past-year substance use** |  |  | |  |  |  | |  |  |  | |  |  |  | |  |  |  | |  |
| Ecstasy | **2.9** | 2.8 | | 3.1 | **32.2** | 29.0 | | 35.4 | **22.9** | 21.2 | | 24.5 | **3.6** | 2.9 | | 4.4 | **0.9** | 0.8 | | 1.0 |
| Meth/amphetamine | **1.3** | 1.2 | | 1.4 | **18.7** | 16.2 | | 21.2 | **8.1** | 7.0 | | 9.1 | **3.9** | 3.2 | | 4.6 | **0.3** | 0.3 | | 0.4 |
| Cocaine | **4.2** | 4.0 | | 4.4 | **41.3** | 38.1 | | 44.6 | **28.8** | 27.1 | | 30.6 | **6.2** | 5.4 | | 7.1 | **1.6** | 1.5 | | 1.7 |
| Hallucinogens | **1.5** | 1.4 | | 1.7 | **20.7** | 18.0 | | 23.5 | **13.9** | 12.5 | | 15.3 | **1.6** | 1.1 | | 2.2 | **0.3** | 0.2 | | 0.4 |
| Inhalants | **1.4** | 1.3 | | 1.5 | **12.7** | 10.3 | | 15.0 | **7.3** | 6.3 | | 8.3 | **1.3** | 0.9 | | 1.7 | **0.7** | 0.7 | | 0.8 |
| Opioids | **2.8** | 2.6 | | 2.9 | **20.3** | 17.6 | | 22.9 | **8.2** | 7.2 | | 9.2 | **5.3** | 4.5 | | 6.1 | **1.9** | 1.7 | | 2.0 |
| **Alcohol use behaviour (AUDIT-C Scale)** |  |  | |  |  |  | |  |  |  | |  |  |  | |  |  |  | |  |
| No risk | **24.6** | 24.2 | | 25.0 | **2.6** | 1.6 | | 3.7 | **2.7** | 2.1 | | 3.3 | **16.9** | 15.5 | | 18.4 | **27.0** | 26.6 | | 27.4 |
| Low risk | **23.9** | 23.6 | | 24.3 | **7.5** | 6.0 | | 9.1 | **10.0** | 9.0 | | 11.0 | **20.7** | 19.3 | | 22.2 | **25.4** | 25.0 | | 25.8 |
| Medium risk | **22.4** | 22.1 | | 22.7 | **13.5** | 11.4 | | 15.5 | **20.2** | 18.8 | | 21.7 | **16.8** | 15.6 | | 18.1 | **23.1** | 22.7 | | 23.5 |
| High risk | **29.1** | 28.7 | | 29.5 | **76.4** | 73.8 | | 78.9 | **67.1** | 65.5 | | 68.8 | **45.5** | 43.7 | | 47.2 | **24.4** | 24.1 | | 24.8 |

AUDIT-C, Alcohol Use Disorders Identification Test Consumption short-form; CI, confidence interval.

**Table S4 Associations between latent class memberships and socio-demographics, health rated correlates and history of past-year substance use with non-user** **as reference group (class 4)**

|  | **Co-use tobacco and cannabis (class 1)** | | | | **Cannabis (class 2)** | | | | **Tobacco (class 3)** | | | |
| --- | --- | --- | --- | --- | --- | --- | --- | --- | --- | --- | --- | --- |
|  | OR | 99.70% CI | | F-statistic, p-value | OR | 99.70% CI | | F-statistic, p-value | OR | 99.70% CI | | F-statistic, p-value |
| **Sex** *(ref: female)* |  |  |  |  |  |  |  |  |  |  |  |  |
| Male | 3.3* | 1.8 | 6.2 | F = 33.6*, p <0.001 | 1.6* | 1.2 | 2.1 | F = 29.2*, p <0.001 | 1.6* | 1.2 | 2.1 | F = 27.9*, p <0.001 |
| **Age group** *(ref: 60+)* |  |  |  |  |  |  |  |  |  |  |  |  |
| 14-29 | 11.7* | 2.0 | 69.3 | F = 5.7*, p = 0.001 | 4.3* | 2.2 | 8.2 | F = 16.3*, p <0.001 | 2.2* | 1.2 | 4.1 | F = 25.4*, p <0.001 |
| 30-39 | 8.0* | 1.4 | 45.6 |  | 3.9* | 2.1 | 7.3 |  | 3.3* | 2.0 | 5.6 |  |
| 40-59 | 7.8* | 1.5 | 40.9 |  | 3.4* | 1.9 | 6.0 |  | 3.4* | 2.2 | 5.3 |  |
| **Marital Status** *(ref: currently married)* |  |  |  |  |  |  |  |  |  |  |  |  |
| Never married | 1.6 | 0.8 | 3.3 | F = 1.8, p = 0.160 | 2.0* | 1.4 | 2.8 | F = 21.0*, p <0.001 | 1.6* | 1.1 | 2.3 | F = 15.6*, p <0.001 |
| Divorced/separated/widowed | 1.4 | 0.5 | 3.7 |  | 1.7* | 1.1 | 2.6 |  | 1.8* | 1.3 | 2.5 |  |
| **Household income** *(ref: 1st quartile: high)* |  |  |  |  |  |  |  |  |  |  |  |  |
| 2nd quartile (high-average) | 2.5* | 1.1 | 5.5 | F = 7.9*, p <0.001 | 1.4 | 1.0 | 2.0 | F = 4.1, p = 0.006 | 2.2* | 1.5 | 3.2 | F = 21.4*, p <0.001 |
| 3rd quartile (low average) | 2.8* | 1.2 | 6.5 |  | 1.4 | 1.0 | 2.1 |  | 2.0* | 1.4 | 3.0 |  |
| 4th quartile (low) | 4.4* | 1.7 | 11.5 |  | 1.5 | 1.0 | 2.4 |  | 3.0* | 2.0 | 4.7 |  |
| **Remoteness** *(ref: major cities)* |  |  |  |  |  |  |  |  |  |  |  |  |
| Inner regional | 0.8 | 0.3 | 1.7 | F = 1.5, p = 0.222 | 1.3 | 0.9 | 1.8 | F = 4.7, p = 0.009 | 1.3 | 0.9 | 1.7 | F = 10.1*, p <0.001 |
| Outer regional, remote or very remote | 1.4 | 0.6 | 3.1 |  | 1.3 | 0.9 | 1.9 |  | 1.7* | 1.2 | 2.3 |  |
| **Highest education attainment** *(ref: bachelor or higher)* |  |  |  |  |  |  |  |  |  |  |  |  |
| High school or less | 1.5 | 0.7 | 3.5 | F = 4.3, p = 0.014 | 1.0 | 0.7 | 1.4 | F = 2.8, p = 0.059 | 2.6* | 1.8 | 3.8 | F = 27.9*, p <0.001 |
| Certificate or diploma | 2.1 | 1.0 | 4.7 |  | 1.3 | 0.9 | 1.7 |  | 2.4* | 1.6 | 3.5 |  |
| **Country of birth** *(ref: outside of Australia)* |  |  |  |  |  |  |  |  |  |  |  |  |
| Australia | 1.2 | 0.5 | 2.7 | F = 0.4, p = 0.512 | 1.1 | 0.8 | 1.5 | F = 0.6, p = 0.436 | 0.8 | 0.6 | 1.2 | F = 2.4, p = 0.116 |
| **Employment** *(ref: currently employed)* |  |  |  |  |  |  |  |  |  |  |  |  |
| Not in labour force | 0.8 | 0.2 | 2.4 | F = 2.6, p = 0.072 | 0.7 | 0.4 | 1.2 | F = 1.8, p = 0.169 | 0.7 | 0.4 | 1.0 | F = 6.8*, p = 0.001 |
| Unemployed | 1.7 | 0.8 | 4.0 |  | 0.9 | 0.5 | 1.7 |  | 1.3 | 0.8 | 2.0 |  |
| **General health** *(ref: excellent)* |  |  |  |  |  |  |  |  |  |  |  |  |
| Very good | 0.9 | 0.3 | 2.1 | F= 3.9, p = 0.004 | 1.3 | 0.8 | 1.8 | F= 2.0, p = 0.088 | 2.3* | 1.2 | 4.2 | F= 21.7*, p <0.001 |
| Good | 1.6 | 0.7 | 3.9 |  | 1.3 | 0.9 | 2.0 |  | 4.1* | 2.3 | 7.6 |  |
| Fair | 2.6 | 0.9 | 7.5 |  | 1.7 | 1.0 | 3.0 |  | 5.5* | 2.8 | 11.0 |  |
| Poor | 1.6 | 0.2 | 13.9 |  | 1.6 | 0.5 | 4.6 |  | 6.1* | 2.6 | 14.7 |  |
| **Psychological distress** *(ref: K10 - low)* |  |  |  |  |  |  |  |  |  |  |  |  |
| K10 *-* moderate | 1.6 | 0.7 | 3.3 | F = 15.8*, p <0.001 | 1.3 | 1.0 | 1.8 | F = 6.1*, p = 0.002 | 1.0 | 0.7 | 1.3 | F = 5.2, p = 0.006 |
| K10 *-* high to very high | 3.7* | 1.8 | 7.6 |  | 1.5* | 1.0 | 2.2 |  | 1.4 | 1.0 | 2.0 |  |
| **Past-year substance use** *(ref: no)* |  |  |  |  |  |  |  |  |  |  |  |  |
| Ecstasy | 2.9* | 1.0 | 8.5 | F = 9.1*, p = 0.002 | 2.8* | 1.3 | 6.1 | F = 15.8*, p <0.001 | 1.6 | 0.6 | 4.4 | F = 1.8, p = 0.184 |
| Meth/amphetamine | 18.9* | 4.5 | 79.4 | F = 36.8*, p <0.001 | 7.7* | 2.0 | 29.3 | F = 20.7*, p <0.001 | 9.6* | 2.8 | 32.4 | F = 30.3*, p <0.001 |
| Cocaine | 7.5* | 3.3 | 17.2 | F = 52.8*, p <0.001 | 5.2* | 3.1 | 8.8 | F = 88.3*, p <0.001 | 3.2* | 1.7 | 6.2 | F = 27.6*, p <0.001 |
| Hallucinogens | 8.5* | 2.2 | 32.4 | F = 22.5*, p <0.001 | 6.1* | 2.0 | 18.7 | F = 23.5*, p <0.001 | 1.8 | 0.4 | 8.2 | F = 1.2, p = 0.269 |
| Inhalants | 1.6 | 0.5 | 5.8 | F = 1.4, p = 0.246 | 1.4 | 0.5 | 3.8 | F = 0.9, p = 0.337 | 1.1 | 0.4 | 3.3 | F = 0.1, p = 0.797 |
| Opioids | 4.0* | 1.6 | 10.0 | F = 20.7*, p <0.001 | 2.0 | 1.0 | 4.1 | F = 8.6, p = 0.003 | 2.0* | 1.0 | 3.8 | F = 10.0*, p = 0.002 |
| **AUDIT-C (Risk of alcohol use disorder)** |  |  |  |  |  |  |  |  |  |  |  |  |
| High risk | 4.3* | 2.0 | 9.0 | F = 33.9*, p <0.001 | 3.1* | 2.3 | 4.2 | F = 135.2*, p <0.001 | 2.0* | 1.5 | 2.6 | F = 59.7*, p <0.001 |

*Significant at the .0029 level, two sided test.

AUDIT-C, Alcohol Use Disorders Identification Test Consumption short-form; CI, confidence interval; OR, odds ratio.

**Table S5. Number and proportion of missing values**

| **Variable** | **Number of missing values** | **Proportion of missing values (%)** |
| --- | --- | --- |
| Sex | 0 | - |
| Marital status | 99 | 0.5 |
| Household income | 6817 | 31.0 |
| Highest education attainment | 1503 | 6.8 |
| Country of birth | 1733 | 7.9 |
| Employment | 1112 | 5.1 |
| General health | 98 | 0.5 |
| K10 psychological distress | 158 | 0.7 |
| Ecstasy | 222 | 1.0 |
| Meth/amphetamine | 181 | 0.8 |
| Cocaine | 226 | 1.0 |
| Hallucinogens | 209 | 1.0 |
| Inhalants | 221 | 1.0 |
| Opioids | 318 | 1.4 |
| AUDIT-C | 139 | 0.6 |

AUDIT-C, Alcohol Use Disorders Identification Test Consumption short-form.
